# Clinically relevant hypomagnesemia during recovery from severe leptospirosis-associated acute kidney injury in a dog treated with continuous renal replacement therapy: a case report

**DOI:** 10.1186/s12917-026-05980-0

**Published:** 2026-10-03

**Authors:** Jaechun Cho, Seongwoo Kang, Sehun Kim, Minsuk Kim, Chul Park

**Affiliations:** 1 May Animal Medical Center, Jeonju, Republic of Korea; 2 Department of Veterinary Internal Medicine, College of Veterinary Medicine, Jeonbuk National University, 79 Gobong-ro, Iksan, Jeonbuk State 54596 Republic of Korea

**Keywords:** Leptospirosis, Acute kidney injury, Hypomagnesemia, Fractional excretion of magnesium, Renal tubular dysfunction, Continuous renal replacement therapy, Dog

## Abstract

**Background:**

Leptospirosis is an important cause of acute kidney injury (AKI) in dogs and commonly induces renal tubular dysfunction. Although electrolyte abnormalities are frequently recognized during the course of leptospirosis, clinically relevant hypomagnesemia associated with neurological manifestations has rarely been reported in dogs. This report describes a dog that developed severe symptomatic hypomagnesemia during recovery from severe leptospirosis-associated AKI treated with continuous renal replacement therapy (CRRT).

**Case presentation:**

A 9 year old, 12 kg spayed female mixed-breed dog was referred for severe leptospirosis-associated AKI complicated by anuria, hyperbilirubinemia, thrombocytopenia, and pulmonary involvement. Blood and urine polymerase chain reaction assays confirmed *Leptospira* spp. infection. Because of persistent anuria and progressive azotemia despite intensive medical management, the dog underwent two continuous renal replacement therapy sessions approximately 15 and 18 h, respectively. Urine production subsequently recovered, and the dog entered a polyuric recovery phase with progressive improvement in azotemia. On day 13 after admission, generalized tremors, hypersalivation, and altered mentation developed concurrently with severe hypomagnesemia (ionized magnesium, 0.24 mmol/L; total magnesium, 1.4 mg/dL). Fractional excretion of magnesium (FEMG) was markedly increased (38.8%), suggesting inappropriate renal magnesium loss despite improving renal function. Intravenous magnesium sulfate administered as a bolus followed by continuous rate infusion resulted in rapid resolution of the neurological signs. At follow-up evaluations performed 11 and 23 days after the hypomagnesemic episode, fractional excretion of magnesium remained increased at 30.2% and 28.4%, respectively, despite improvement in serum creatinine concentration, supporting persistent impairment of renal tubular magnesium handling during recovery.

**Conclusions:**

This case demonstrates that clinically significant hypomagnesemia may develop during the polyuric recovery phase of severe leptospirosis-associated AKI in dogs, even after apparent improvement in renal function. Serial measurements of fractional excretion of magnesium supported continued renal magnesium loss during recovery and emphasized the importance of monitoring magnesium concentrations in dogs with leptospirosis, particularly when neurological abnormalities develop.

## Background

Leptospirosis is an important zoonotic disease that can cause multisystemic injury in dogs. Among the affected organs, the kidneys are a major target, and renal involvement commonly manifests as acute kidney injury (AKI) [1–5]. 

Renal injury is characterized predominantly by acute tubulointerstitial nephritis, which can impair tubular reabsorptive function and electrolyte disturbances, including abnormalities in sodium and potassium homeostasis [6, 7]. 

In humans, renal magnesium wasting and symptomatic hypomagnesemia have been reported during the polyuric phase of leptospirosis-associated AKI and are considered consequences of tubular dysfunction [8–10]. 

However, clinically relevant hypomagnesemia secondary to leptospirosis has rarely been described in dogs, and objective assessment of renal magnesium handling using serial fractional excretion of magnesium (FEMG) measurements has not been reported [11]. 

Here, we describe a dog with severe leptospirosis-associated AKI that developed clinically relevant hypomagnesemia accompanied by neurological manifestations during the polyuric recovery phase, with serial fractional excretion of magnesium measurements suggesting continued renal magnesium loss despite improving renal function.

## Case presentation

A 9-year-old, 12-kg, spayed female mixed-breed dog was referred to a referral hospital with a 1-day history of anorexia, lethargy, and dark yellow urine. The dog was housed primarily outdoors in a yard and had not been vaccinated against leptospirosis.

On presentation, the dog was depressed and moderately dehydrated, with icteric sclera and mucous membranes, a rectal temperature of 39.1 °C, a heart rate of 140 beats/min, systolic arterial blood pressure of 110 mmHg, and panting respiration. Harsh lung sounds were auscultated bilaterally. Neurological examination was unremarkable.

Initial hematologic evaluation revealed severe thrombocytopenia (13 × 10⁹/L) without anemia (hematocrit, 40.9%). Serum biochemistry analysis demonstrated severe azotemia (blood urea nitrogen, 103 mg/dL; creatinine, 7.1 mg/dL), hyperphosphatemia (8.6 mg/dL), marked elevation of alkaline phosphatase activity (1,242 U/L), hypoalbuminemia (2.7 g/dL), and markedly increased C-reactive protein concentration (150 mg/L) **(**Table 1**).** Coagulation testing revealed a markedly prolonged activated partial thromboplastin time (174 s), a mildly prolonged prothrombin time (18 s), and a markedly increased D-dimer concentration (4,664 ng/mL). Serial platelet counts and coagulation profiles are presented in Table 2. Symmetric dimethylarginine (SDMA) exceeded the measurable range. Urinalysis showed glucosuria (1+) despite normoglycemia and bilirubinuria (1+).

**Table 1:** Serial clinicopathological findings in the present case

Variable	Reference interval	Admission Day 1	First CRRT Day 2	Second CRRT Day 4	Post-CRRT Day 5	Peak polyuria Day 7	Hypomagnesemic episode Day 13	Discharge Day 20	Follow-up 1 Day 24	Follow-up 2 Day 36
HCT (%)	37.3–61.7	40.9	29.9	20.4	18.9	25.9	35.3	29	26	26
PLT (×10⁹/L)	148–484	13	12	–	9	21	25	15	38	79
Neu (×10⁹/L)	2.95–11.64	6.4	8.1	7.5	11	40.3	45	18	38	16
pH	7.31–7.46	7.4	7.45	7.4	7.38	7.35	7.48	7.36	7.42	7.45
Na⁺ (mmol/L)	145–151	137	136	138	138	131	140	149	142	144
K⁺ (mmol/L)	3.9–5.1	5.1	5.9	6.7	6.2	5.3	4.7	3.1	3	3.7
Cl⁻ (mmol/L)	110–119	104	107	112	110	102	105	110	108	110
iCa (mmol/L)	1.16–1.40	1.22	1.15	1.26	1.29	1.32	1.14	1.31	1.11	1.07
iMg (mmol/L)	0.40–0.60	0.4	0.52	0.56	0.51	0.44	0.24	0.38	0.34	0.3
Lac (mmol/L)	0.3–2.5	1.7	2.5	0.2	0.2	0.3	3.6	6.3	6.2	3.5
TBil (mg/dL)	0.1–0.5	0.5	2.1	10.3	10.8	14.8	5.9	2.9	2.6	1.2
BUN (mg/dL)	9.2–29.2	103	165	133	130	178	92	70	73	45
Crea (mg/dL)	0.4–1.4	7.1	9.2	6.6	5.7	7.8	4.1	3.4	2.8	3.2
Phos (mg/dL)	1.9–5.0	8.6	13.7	8.9	9.3	11.3	10.4	9.9	7.9	6
tCa (mg/dL)	9.3–12.1	–	–	–	–	–	–	13	10.1	10.3
tMg (mg/dL)	1.5–2.7						1.4	1.8	1.6	1.5
AST (U/L)	17–44	99	411	108	–	–	129	–	75	63
ALT (U/L)	17–78	55	–	–	–	–	177	–	194	278
ALP (U/L)	47–254	1242	–	–	–	–	3800	–	3890	3560
GGT (U/L)	5–14	–	–	–	–	–	–	–	83	38
ALB (g/dL)	2.6–4.0	2.7	–	–	–	–	2.7	–	3.2	2.9
TP (g/dL)	5.0–7.2	6	–	–	–	–	5.7	–	6.3	5.8
CRP (mg/L)	0–9	150	92	–	–	–	113	–	31	12

An en dash indicates that the analyte was not measured at that time point. Reference intervals were taken from the analyzers used for this patient

*Abbreviations*: *ALB* Albumin, *ALP* Alkaline phosphatase, *ALT* Alanine aminotransferase, *AST* Aspartate aminotransferase, *BUN* Blood urea nitrogen *Cl*⁻ Chloride, Crea, creatinine, *CRP* C-reactive protein, *CRRT* Continuous renal replacement therapy, *GGT* γ-glutamyl transferase *HCT* Hematocrit, *iCa* ionized calcium, *iMg* ionized magnesium, *K*⁺ potassium, *Lac* lactate,* Na*⁺ sodium *Neu* absolute neutrophil count, Phos, phosphorus, *PLT* Platelet count *TBil* Total bilirubin, *tCa* Total calcium, *TP* Total protein

**Table 2:** Serial changes in platelet count and coagulation variables during hospitalization

Variable	Reference range	Day 1	Day 2	Day 3	Day 4	Day 8	Day 13	Day 19	Day 24	Day 36
Platelet count, ×10³/µL	148–484	13	12	2	0	21	25	32	38	79
PT, s	14–20	18	—	—	—	—	22	—	18	—
APTT, s	94–123	174	—	—	—	—	261	—	123	—
D-dimer, ng/mL	0–300	4,664	—	—	—	—	1,663	—	870	—

An em dash indicates that the variable was not measured. Reference intervals were obtained from the analyzers used for this patient

*Abbreviations: APTT*Activated partial thromboplastin time,*PT*Prothrombin time

**Fig. 1:**
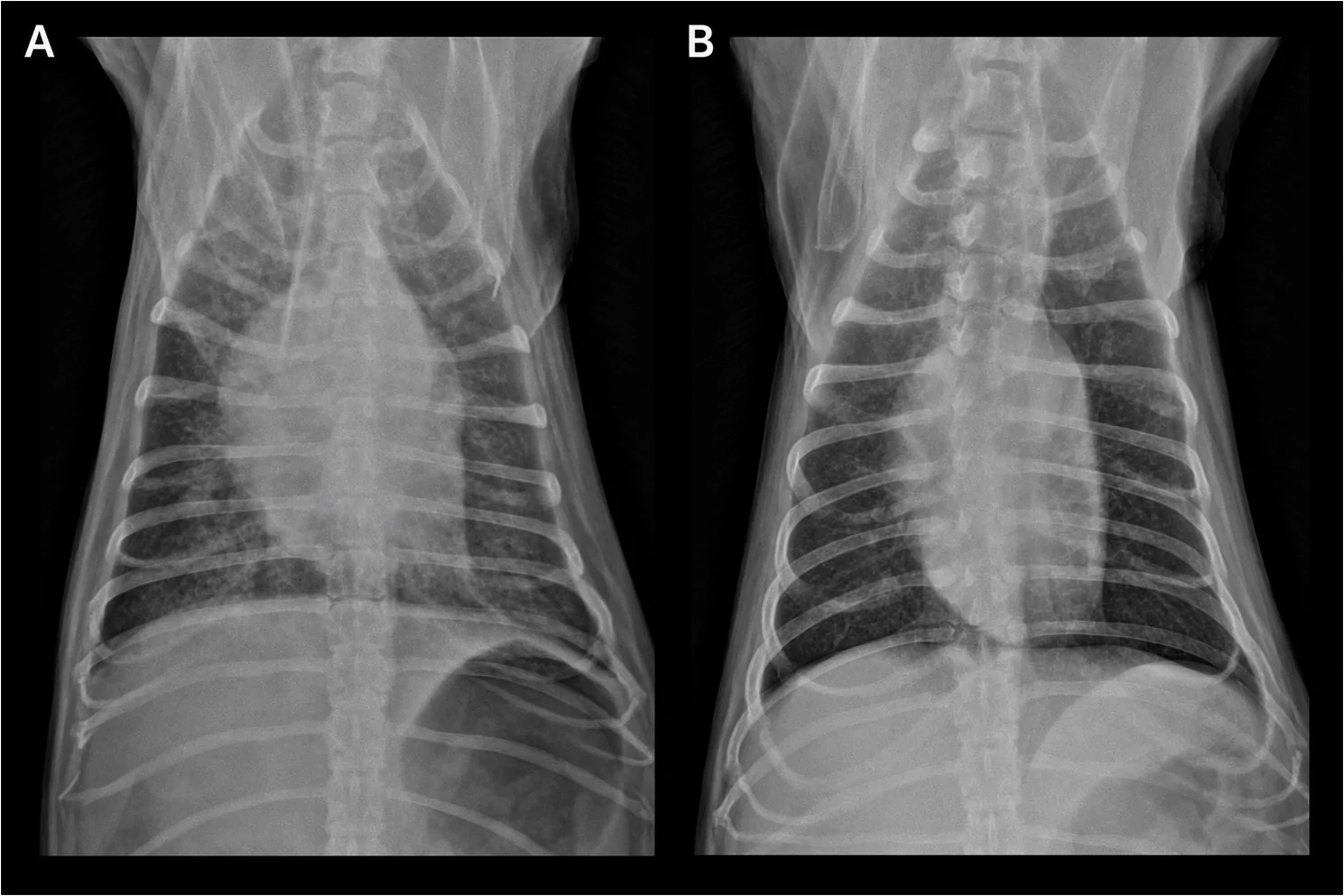
Serial thoracic radiographs obtained during hospitalization. **A** Thoracic radiograph obtained at presentation showing diffuse bilateral interstitial-to-alveolar pulmonary infiltrates, predominantly involving the caudodorsal lung fields. **B** Follow-up thoracic radiograph showing marked improvement in the previously observed pulmonary infiltrates

**Fig. 2:**
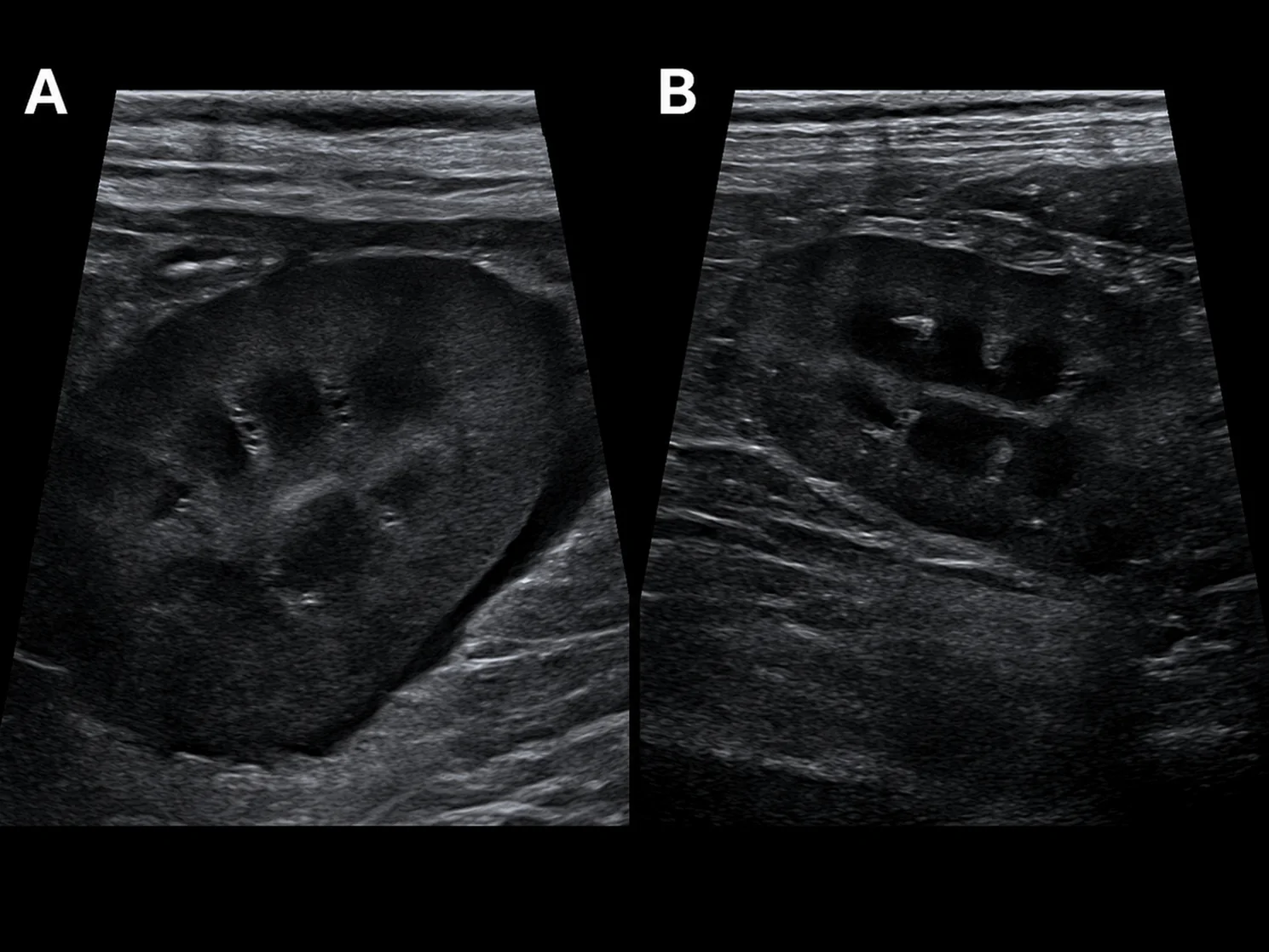
Serial ultrasonographic images of the left kidney. **A** Ultrasonographic image obtained during hospitalization showing mild perirenal fluid accumulation around the left kidney. **B** Follow-up ultrasonographic image showing resolution of the perirenal fluid accumulation

Thoracic radiographs obtained at presentation revealed diffuse bilateral interstitial-to-alveolar pulmonary infiltrates, predominantly involving the caudodorsal lung fields **(**Fig. 1A**)**. Abdominal ultrasonography demonstrated mild perirenal fluid accumulation around the left kidney **(**Fig. 2A**).** Based on the dog’s outdoor lifestyle, compatible clinical signs, severe AKI with hepatic involvement, and these imaging findings, leptospirosis was considered the primary differential diagnosis.

Supportive treatment was initiated immediately after admission and included intravenous fluid therapy, oral doxycycline (5 mg/kg q12h), and intravenous ampicillin-sulbactam (22 mg/kg q8h). Blood and urine samples were submitted to a veterinary diagnostic laboratory (GreenVet, Republic of Korea) for Leptospira spp. real-time PCR testing. The blood sample was analyzed as part of a multiplex canine anemia/vector-borne pathogen PCR panel, whereas the urine sample was analyzed separately for Leptospira spp. by real-time PCR. Despite intensive medical management, the dog became anuric within 24 h of hospitalization, accompanied by progressive azotemia and persistent hyperphosphatemia. Continuous renal replacement therapy (CRRT) was therefore initiated on hospital day 2. Treatment was performed using an 8-Fr, 20-cm double-lumen catheter placed in the right jugular vein for vascular access. A Fresenius multiFiltrate system (Fresenius Medical Care AG & Co. KGaA, Bad Homburg, Germany) was used in pediatric continuous venovenous hemodialysis (CVVHD) mode with a polysulfone dialyzer (Ultraflux AV paed, Fresenius Medical Care AG & Co. KGaA, Bad Homburg, Germany). The extracorporeal circuit was primed with 180 mL of crossmatch-compatible whole blood before each treatment session. Blood flow was maintained at 50–60 mL/min, and dialysate flow was set at 800–850 mL/h. A potassium-free multiBic solution (Fresenius Medical Care AG & Co. KGaA, Bad Homburg, Germany) containing sodium 140 mmol/L, potassium 0 mmol/L, calcium 1.5 mmol/L, magnesium 0.5 mmol/L, chloride 109 mmol/L, bicarbonate 35 mmol/L, and glucose 5.55 mmol/L was used as the dialysate. No separate replacement fluid was administered. No additional magnesium supplementation was provided during either CRRT session. Net ultrafiltration was initially set at 10 mL/h and adjusted according to the degree of fluid overload. Because of severe thrombocytopenia, anticoagulation was initially performed without heparin and, when required, unfractionated heparin was administered by continuous infusion at up to 5 U/kg/h. The prescribed treatment target was a urea reduction ratio of approximately 80%, with a target BUN concentration of approximately 30 mg/dL. The first CRRT session lasted approximately 15 h. Because anuria persisted approximately 8 h after completion of the first treatment, a second CRRT session using a similar prescription was initiated on hospital day 4 and continued for approximately 18 h. During treatment, blood pressure, urine output, electrolyte and acid–base status, and extracorporeal circuit pressures were monitored serially.

**Fig. 3:**
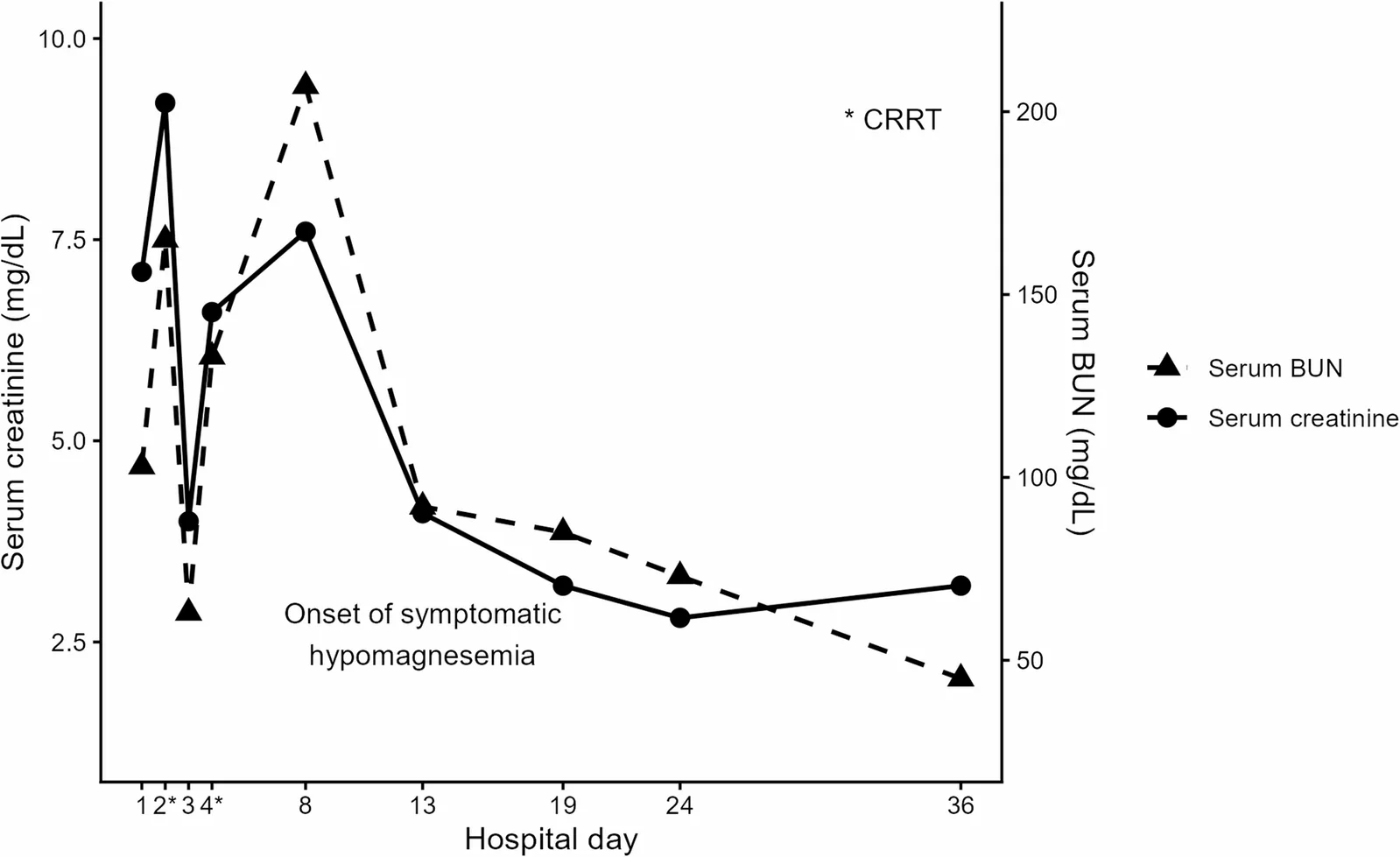
Serial changes in blood urea nitrogen and serum creatinine concentrations during hospitalization and follow-up Changes in blood urea nitrogen and serum creatinine concentrations are shown over time. The periods of continuous renal replacement therapy and the onset of symptomatic hypomagnesemia are indicated.BUN, blood urea nitrogen; CRRT, continuous renal replacement therapy

On hospital day 4, both blood and urine polymerase chain reaction (PCR) assays returned positive for *Leptospira* spp., confirming the diagnosis of leptospirosis. Following the second CRRT session, urine production gradually recovered, increasing from 2.14 mL/kg/h on hospital day 5 to 9.4 mL/kg/h on day 6 and 13.0 mL/kg/h on day 7, consistent with the polyuric recovery phase. Concurrently, serum creatinine and blood urea nitrogen concentrations gradually decreased, although hyperphosphatemia persisted throughout the recovery period **(**Fig. 3**).**

During the polyuric recovery phase, intravenous fluid therapy was adjusted according to urine output and hydration status, and potassium supplementation was provided as needed based on serial electrolyte measurements. Nutritional support was provided enterally as tolerated. No vomiting or diarrhea was observed during hospitalization. No additional magnesium supplementation was administered before the onset of symptomatic hypomagnesemia. On hospital day 13, despite continued improvement in azotemia, the dog acutely developed generalized tremors, hypersalivation, and marked altered mentation. Point-of-care electrolyte analysis demonstrated severe ionized hypomagnesemia (0.24 mmol/L), which was accompanied by a decreased serum total magnesium concentration (1.4 mg/dL). At the time of the neurological episode, ionized calcium was mildly decreased (1.14 mmol/L), lactate was increased (3.6 mmol/L), and systolic blood pressure was 150 mmHg. Blood glucose measured later that day was 131 mg/dL. Serum and urine magnesium and creatinine concentrations used for FEMG calculation were measured using a dry chemistry analyzer (FDC NX700; FUJIFILM, Tokyo, Japan) from paired samples collected contemporaneously. FEMG was calculated as (urine magnesium × serum creatinine) / (0.7 × serum magnesium × urine creatinine) × 100 [12, 13]. At each FEMG assessment, paired serum and urine samples were collected before magnesium supplementation was administered or initiated. FEMG was markedly increased at 38.8%, suggesting inappropriate urinary magnesium loss despite improving renal function. Serial ionized magnesium concentrations and FEMG values are presented in Table 3.

**Table 3:** Serial ionized magnesium concentrations and fractional excretion of magnesium during hospitalization and follow-up

Variable	Day 1	Day 2	Day 3	Day 4	Day 8	Day 13	Day 19	Day 24	Day 36
iMg (mmol/L)	0.40	0.52	0.46	0.56	0.40	0.24	0.40	0.34	0.30
Serum Mg (mg/dL)						1.4		1.6	1.5
Serum creatinine (mg/dL)						4.1		2.8	3.2
Urine Mg (mg/dL)						3.6		4.5	4.8
Urine creatinine (mg/dL)						39		38	52
FEMg (%)	—	—	—	—	—	38.8	—	30.2	28.4

*Abbreviations*:* iMg* ionized magnesium, *FEMg* fractional excretion of magnesium, *Mg* magnesium

Because of clinically significant symptomatic hypomagnesemia, 10% magnesium sulfate diluted in 0.9% saline was administered intravenously as an initial bolus over 5 min (approximately 0.05 mmol/kg Mg²⁺ [0.10 mEq/kg]), followed by continuous rate infusion in 0.9% saline for approximately 24 h (approximately 1.3 mEq/kg/day Mg²⁺). The generalized tremors, hypersalivation, and altered mentation improved rapidly following magnesium supplementation, with a progressive increase in ionized magnesium concentration and no clinically apparent adverse effects.

The dog’s clinical condition continued to improve. A urine PCR assay for *Leptospira* spp. performed on hospital day 19 was negative. Follow-up thoracic radiographs showed marked improvement in the previously observed pulmonary infiltrates **(**Fig. 1B**)**, and ultrasonography demonstrated resolution of the perirenal fluid accumulation around the left kidney **(**Fig. 2B**)**. The dog was discharged on hospital day 20 with improved appetite, activity, and urine output.

At the first follow-up evaluation on hospital day 24, 4 days after discharge and 11 days after the hypomagnesemic episode, the dog remained clinically stable without recurrence of neurological signs. Serum creatinine concentration had further decreased to 2.86 mg/dL, ionized magnesium concentration was 0.34 mmol/L, and serum total magnesium concentration was 1.6 mg/dL. However, FEMG remained increased at 30.2%.

At the second follow-up evaluation on hospital day 36, 16 days after discharge and 23 days after the hypomagnesemic episode, serum creatinine concentration was 3.23 mg/dL, ionized magnesium concentration remained decreased at 0.30 mmol/L, serum total magnesium concentration was 1.5 mg/dL, and FEMG remained increased at 28.4%. These findings supported persistent impairment of renal tubular magnesium handling despite substantial clinical improvement; renal recovery remained incomplete, as serum creatinine remained above the reference interval during follow-up. Oral magnesium supplementation was subsequently initiated using a magnesium oxide–containing supplement at a labeled elemental magnesium dose of 200 mg once daily (approximately 16.7 mg/kg/day), and the dog remained clinically stable without recurrence of neurological signs. The major clinical events, treatments, and follow-up findings are summarized in Table 4.

**Table 4:** Timeline of major clinical events, treatment, and follow-up

Time	Event
Day 1	Presentation with severe leptospirosis-associated AKI and supportive treatment initiated.
Day 2	First CRRT session, lasting approximately 15 h, for persistent anuria and progressive azotemia.
Day 4	Blood and urine PCR positive for Leptospira spp.; second CRRT session, lasting approximately 18 h, initiated.
Days 5–7	Polyuric recovery phase with increasing urine output and improving azotemia.
Day 13	Symptomatic hypomagnesemia (iMg, 0.24 mmol/L; FEMg, 38.8%); Intravenous magnesium administered with rapid resolution of neurological signs.
Days 19–20	Urine PCR negative; pulmonary infiltrates and perirenal fluid improved; discharged.
Day 24	First follow-up: iMg, 0.34 mmol/L; FEMg, 30.2%; no recurrence of neurological signs.
Day 36	Second follow-up: iMg, 0.30 mmol/L; FEMg, 28.4%; oral magnesium supplementation initiated.
Follow-up	Clinically stable without recurrence of neurological signs.

*Abbreviations*: *AKI* Acute kidney injury *CRRT* Continuous renal replacement therapy *FEMg* Fractional excretion of magnesium, *iMg* ionized magnesium, *PCR* polymerase chain reaction

## Discussion

This report describes clinically relevant hypomagnesemia that developed during the polyuric recovery phase of severe leptospirosis-associated AKI in a dog treated with CRRT [14, 15]. The severity of disease was evidenced by AKI requiring CRRT, marked hyperbilirubinemia, pulmonary involvement, and coagulation abnormalities, consistent with the multisystemic manifestations reported in dogs with severe leptospirosis [16–18]. Although renal tubular dysfunction and urinary electrolyte wasting are recognized features of leptospiral AKI, symptomatic hypomagnesemia has not been well characterized in dogs.

In a prospective study of dogs with leptospirosis-associated AKI, glucosuria and increased urinary potassium excretion supported the presence of leptospirosis-associated tubular dysfunction [7]. Although serum magnesium concentration and fractional excretion of magnesium were also evaluated, hypomagnesemia was not identified, and magnesium-related variables did not differ significantly from those in dogs with AKI of other etiologies. Importantly, urine chemistry was assessed only at hospital admission, and therefore delayed magnesium wasting during renal recovery may not have been captured. Alternative contributors to hypomagnesemia, including gastrointestinal losses, hypercalcemia, diuretic administration, and magnesium removal during CRRT, were also considered; however, none appeared sufficient to explain the severity and persistence of hypomagnesemia in this case [19, 20]. 

In the present case, severe hypomagnesemia was identified during the polyuric recovery phase and was accompanied by generalized tremors, hypersalivation, and altered mentation despite progressive improvement in azotemia. The neurological signs resolved promptly following intravenous magnesium supplementation. The temporal association between severe hypomagnesemia and the onset of neurological signs, together with their rapid improvement following magnesium supplementation, supports a possible contribution of hypomagnesemia to the neurological manifestations. However, concurrent metabolic and systemic abnormalities associated with severe AKI and leptospirosis may also have contributed to these signs. Serial measurements of FEMG remained markedly increased during follow-up, supporting persistent impairment of renal tubular magnesium reabsorption even as glomerular function improved. Magnesium is predominantly reabsorbed in the thick ascending limb of the loop of Henle and the distal convoluted tubule [21, 22]. Therefore, renal tubular injury can impair magnesium reabsorption and promote inappropriate urinary magnesium loss [23, 24]. 

In humans with leptospirosis, inappropriate renal magnesium wasting has been identified as an important mechanism of hypomagnesemia. A prospective study documented hypermagnesuria in most affected patients, with markedly increased FEMG in those who developed hypomagnesemia and AKI, while a separate case report demonstrated severe symptomatic hypomagnesemia accompanied by persistently elevated FEMG [8, 9]. Although veterinary-specific reference thresholds have not been established, an FEMG > 2% in hypomagnesemic human patients is considered suggestive of inappropriate renal magnesium wasting [11]. 

In this patient, severe hypomagnesemia developed after restoration of urine output and coincided with markedly increased FEMG despite progressive improvement in azotemia. FEMG was 38.8% at the onset of neurological signs and remained elevated at 30.2% and 28.4% on two subsequent assessments. These findings indicate persistent impairment of tubular magnesium reabsorption despite improving glomerular function and suggest that a decrease in serum creatinine concentration should not be interpreted as evidence of complete tubular recovery.

This report has several limitations. First, renal tubular injury was not confirmed histopathologically, and the proposed mechanism of impaired tubular magnesium handling was inferred from clinicopathological findings and serial FEMG measurements. Second, tubular injury biomarkers, such as urinary neutrophil gelatinase-associated lipocalin, were not measured; therefore, persistent tubular injury could not be directly confirmed. Third, FEMG was calculated from spot urine samples rather than timed urine collections, which may have limited precise quantification of urinary magnesium excretion.

In conclusion, this case describes clinically relevant hypomagnesemia that developed during the polyuric recovery phase of severe leptospirosis-associated acute kidney injury in a dog. Serial FEMG measurements suggested persistent impairment of tubular magnesium handling despite improving renal function. This report highlights hypomagnesemia as a potentially overlooked complication during recovery from canine leptospirosis and emphasizes the importance of considering magnesium imbalance in dogs that develop neurological signs during recovery from AKI.

## Data Availability

All data generated or analyzed during this case report are included in this published article. Additional clinical data are available from the corresponding author upon reasonable request.

## Abbreviations

AKI: Acute kidney injury
aPTT: Activated partial thromboplastin time
BUN: Blood urea nitrogen
CRP: C-reactive protein
CRRT: Continuous renal replacement therapy
FEMG: Fractional excretion of magnesium
iMg: Ionized magnesium
PCR: Polymerase chain reaction
PT: Prothrombin time
SDMA: Symmetric dimethylarginine
